# Use of human bone marrow mesenchymal stem cells immortalized by the expression of telomerase in wound healing in diabetic rats

**DOI:** 10.1590/1414-431X2021e11352

**Published:** 2021-09-03

**Authors:** G.L. Flores Luna, T.L. Oehlmeyer, G. Brandão, P. Brassolatti, J. Tosta, L.S. Goto, L. de Avó, A.M. de Oliveira Leal

**Affiliations:** 1 Post-Graduate Program in Biotechnology, Laboratório de Investigação Endócrina Metabólica, Departamento de Medicina, Universidade Federal de São Carlos, São Carlos, SP, Brasil; 2 Laboratório de Inflamação e Doenças Infecciosas, Departamento de Morfologia e Patologia, Universidade Federal de São Carlos, São Carlos, SP, Brasil; 3 Laboratório Nacional de Biociências, Centro Nacional de Pesquisa em Energia e Materiais, Campinas, SP, Brasil; 4 Departamento de Medicina, Universidade Federal de São Carlos, São Carlos, SP, Brasil

**Keywords:** Cell therapy, Diabetes mellitus, Mesenchymal stem cells, Wound, Animal model

## Abstract

Diabetes mellitus is associated with neural and micro- and macrovascular complications. Therapeutic options for these complications are limited and the delivery of mesenchymal stem cells into lesions have been reported to improve the healing process. In this work, the effects of the administration of a lineage of human bone marrow mesenchymal stem cells immortalized by the expression of telomerase (hBMSC-TERT) as a potential therapeutic tool for wound healing in diabetic rats were investigated. This is the first description of the use of these cells in diabetic wounds. Dorsal cutaneous lesions were made in streptozotocin-induced diabetic rats and hBMSC-TERT were subcutaneously administered around the lesions. The healing process was evaluated macroscopically, histologically, and by birefringence analysis. Diabetic wounded rats infused with hBMSC-TERT (DM-TERT group) and the non-diabetic wounded rats not infused with hBMSC-TERT (CW group) had very similar patterns of fibroblastic response and collagen proliferation indicating improvement of wound healing. The result obtained by birefringence analysis was in accordance with that obtained by the histological analysis. The results indicated that local administration of hBMSC-TERT in diabetic wounds improved the wound healing process and may become a therapeutic option for wounds in individuals with diabetes.

## Introduction

Chronic hyperglycemia due to diabetes mellitus (DM) causes neural and micro- and macro-vascular complications. Amongst these complications, impaired wound healing and diabetic foot are health challenges. In individuals with DM, the physiological sequence of wound healing is disrupted, resulting in a disorganized process in which different areas of the lesion remain in different stages (1,2). More than 100 abnormalities have been described in the diabetic wound healing process and their combined actions result in the disruption of the finely ordered sequence of events leading to the reconstitution of cutaneous integrity. Such changes involve angiogenic responses (3), quantity of granulation tissue, keratinocytes, fibroblast, and macrophage activation, migration and proliferation (3,4), accumulation of extracellular matrix components and their remodeling (5,6), and the complex interaction between the wound and the organism mediated by cytokines, chemokines, and other signaling proteins (7,8).

The scarcity of effective therapeutic options has stimulated new research looking for alternative therapies, such as the use of stem cells (9,10). Stem cell therapy has been demonstrated to be a potential novel therapy for wound healing. As a highlight, mesenchymal stem cells (MSCs) have been studied due to their anti-inflammatory actions and ability to replicate and differentiate into a variety of mesenchymal tissue types (adipose tissue, cartilage, and bone) (11). These cells can migrate to injured sites, where they might act secreting trophic and paracrine mediators, and interact with the immune system cells to induce a more anti-inflammatory phenotype by modulating the immune cells’ responses (12,13). In addition, they might promote neoangiogenesis by increasing the secretion of angiogenic factors and the secretion of collagen by the local fibroblasts (14,15). Therefore, the use of MSCs is an attractive therapeutic strategy that can modify the microenvironment close to wounded areas contributing to tissue repair and regeneration (16-18).

However, the limited useful life of MSCs is a problem that prevents their use for therapeutic application. The finite cell life span is reported in approximately 38 population duplications, at which time these cells no longer divide (19). An alternative solution to this problem may be the use of MSCs modified by telomerase reverse transcriptase (TERT). TERT is a subunit of the telomerase complex catalytic protein and its expression prevents shortening of telomeres at each cell cycle, which makes the cell capable of infinitely expanding and maintaining its primary specification characteristics. The immortalization mechanism was described in the study by Mihara et al. (20) where primary mesenchymal cells collected from the bone marrow of a healthy donor were cultured and subsequently infected with a retroviral vector that contained the hTERT genes and green fluorescent protein (GFP). The stability acquired in this process enables cell proliferation, which favors its application and use in stem cell therapies (20-22).

Thus, the objective of the present study was to analyze the effects of a lineage of human bone marrow mesenchymal stem cells immortalized by the enforced expression of the enzyme telomerase (hBMSC-TERT) in a model of diabetic wound in rats.

## Material and Methods

### Animal care and experimental groups

Male Wistar rats were provided by the University of São Paulo (Ribeirão Preto, SP, Brazil) and maintained in individual polypropylene cages under controlled temperature, humidity, and lighting (12 h light/dark cycle), with food and water supplied *ad libitum*. The study was approved by the Animal Ethics Committee of Federal University of São Carlos (CEUA 97150416) and was carried out in agreement with the Guide for the Care and Use of Laboratory Animals (Institute of Laboratory Animal Resources, National Academy of Sciences, USA) and the principles of the Brazilian College of Animal Experimentation.

After 7 days of acclimation, animals (225±25 g) were randomly assigned to 4 groups: control rats wounded (CW, n=8), non-diabetic rats wounded and infused with hBMSC-TERT (C-TERT, n=10), diabetic rats wounded (DMW, n=8), and diabetic rats wounded and infused with hBMSC-TERT (DM-TERT, n=8). The diabetic rats received streptozotocin (STZ) (60 mg/kg, *ip*; Sigma-Aldrich, USA) and the non-diabetic rats received vehicle (sodium citrate, *ip*). Diabetes was checked 7 days after STZ injection and confirmed when rats were hyperglycemic (blood glucose >250 mg/dL) (23). Body mass and fasting glycemia were determined weekly. Blood samples were collected from the tail vein and glycemia was measured using an Accu-Check glucose meter (Roche Diagnostic, USA).

### Excision wound model in rats

For diabetic rats, the wounds were made 5 weeks after the rats were considered diabetic. Equivalent wounds were made on the same day in non-diabetic animals. The animals were anesthetized with ketamine (90 mg/kg) and xylazine (13 mg/kg) and excisional wounds were made using a 6.0-mm punch on the dorsal skin, as previously described (24). The wound healing process was followed by digital photographs for 21 days and analyzed by ImageJ software (NIH, USA).

### Cell culture procedures

The mesenchymal stem cells hBMSC-TERT used were kindly provided by the Regional Hemotherapy Center of the School of Medicine of Ribeirão Preto, University of São Paulo, Brazil, and immortalized by the expression of telomerase reverse transcriptase. Cell stocks were provided as -80°C remainders that were cultivated in alpha minimum essential medium (α-MEM; Gibco, USA) containing 15% fetal bovine serum (FBS) (HyClone^®^, Thermo Scientific, Canada), HEPES (Sigma-Aldrich), sodium bicarbonate (Sigma-Aldrich), L-glutamine (Sigma-Aldrich), sodium pyruvate, and penicillin/streptomycin (Gibco), in standard polystyrene bottles of 25 and 75 cm^2^ at 37°C and 5% CO_2_ atmosphere. Cells were detached by trypsin (Gibco) and washed three times with sterile phosphate buffer solution (PBS) before use in the experiments.

### Differentiation analysis

The multipotency was tested by inducing hMSC-TERTs differentiation using StemPro osteogenesis, adipogenesis, and chondrogenesis differentiation kits (Life Technologies, USA) in 24-well plates and following the manufacturer's instructions.

### Multiple administrations of hBMSC-TERT

**Figure 1:**
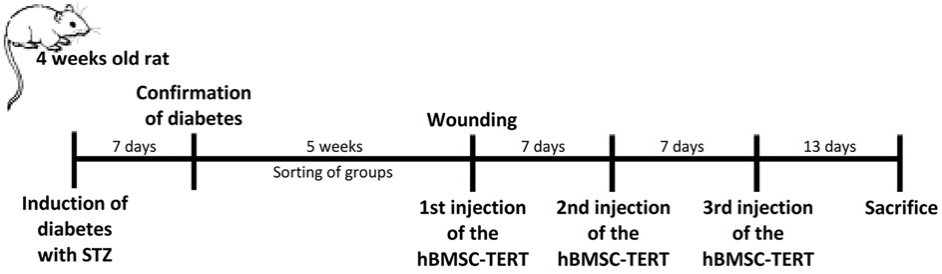
Experimental groups and study design. Five weeks after the confirmation of diabetes, skin lesions were performed and, immediately afterwards, an intracutaneous infusion of phosphate buffer solution (control group and diabetic rats wounded) or 1×10^6^ human bone marrow mesenchymal stem cells immortalized by the expression of telomerase (hBMSC-TERT) (C-TERT and DM-TERT groups) was performed. Fasting glycemia was determined weekly and photographic assessments of the wounds were performed on days 0, 3, 7, 14, and 21 days after the first infusion. STZ: streptozotocin.

The hBMSC-TERT cells at passage 5 were used for multiple infusions in diabetic and non-diabetic rats. The C-TERT and DM-TERT groups received three intracutaneous infusions of 1×10^6^ hBMSC-TERT cells resuspended in 500 µL of PBS around their wounds with a one-week interval. The CW and DMW groups received PBS infusions of 500 µL at the same sites (Figure 1).

### Euthanasia

The animals were euthanized on the 13th day after the last hBMSC-TERT infusion and skin samples were collected for further analysis.

### Histological analysis

After collection, the skin samples were longitudinally cut and fixed in 10% buffered formalin, dehydrated through graded ethanol passages, cleared in xylene, and embedded in paraffin wax (25). Skin sections (5-µm thick) were cut from the paraffin-embedded specimens and stained with hematoxylin-eosin (HE, Merck, Germany). For this analysis, three sections of each slide and 5 slides of each animal were analyzed using a Zeiss Axioshop 2 microscope, under 400× magnification (Carl Zeiss, Germany). For the semi-quantitative analyses, a qualified pathologist used the following scale proposed by De Mayo et al. (26) to estimate the degree of leukocyte infiltration, fibroblastic response, and collagen proliferation: 0) absent (no apparent inflammatory infiltration, fibroblastic response, or collagen proliferation); 1) mild inflammatory response or proliferation findings (<10% of the area covered by inflammatory cells, fibroblastic response, or collagen proliferation); 2) moderate inflammatory response or proliferation findings (10 to 50% of the area covered by inflammatory cells, fibroblastic response, or collagen proliferation); and 3) severe inflammatory response (above 50% of the area covered by inflammatory cells, fibroblastic response, or collagen proliferation). All analyses were performed by an evaluator blinded to the experimental groups.

### Birefringence analysis

The histological slides were stained by the picrosirius-red method and analyzed under a polarized light microscope (Leica, Germany) to evaluate the organization of collagen fibers. For this purpose, three consecutive fields of each sample were photographed with the assistance of a camera attached to the polarized light microscope at a magnification of 400×. The corresponding values were measured for each field, resulting in the birefringence amount of collagen fibers. Quantitative analysis was performed using the Color Deconvolution function of ImageJ 1.37 software to evaluate the percentage of reddish-orange coloration (27).

### Statistical analysis

Data are reported as means±SD. One-way ANOVA was used to analyze the histological parameters. The Tukey test was used for *post hoc* analysis. In all tests, the statistical significance was set at 0.05.

## Results

### Differentiation analysis

**Figure 2:**
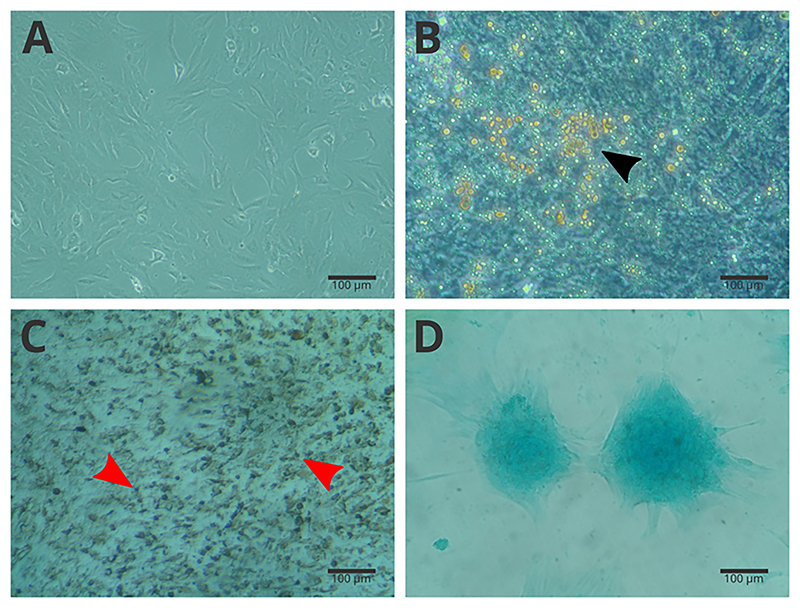
Human bone marrow mesenchymal stem cells immortalized by the expression of telomerase (hBMSC-TERT). Passage five plastic-adherent cells cultured in α-MEM supplemented with 10% fetal bovine serum (**A**) and differentiated into adipogenic lineages identified by Sudan II (0.2%) (**B**), osteogenic lineages identified by von Kossa technique (**C**), and chondrogenic lineages identified by Alcian blue (1%) (**D**). Scale bar 100 μm.

The hBMSC-TERT cells were successfully differentiated into adipocytes, osteocytes, and chondrocytes indicating that they maintained their multipotent properties (Figure 2).

### Body weight and fasting glycemia

The body weight of the DMW and DM-TERT groups was significantly lower than CW and C-TERT animals, whereas fasting glycemia was increased, beginning at one-week post-induction of diabetes until the end of the experimental period. There was no difference among the DM groups (Table 1).

**Table 1:** Body mass (g) and fasting glycemia (mg/dL) pre-diabetes mellitus (DM) induction, pre-infusion of mesenchymal stem cells (MSCs), and pre-sacrifice of animals.

Variable/groups	Pre-DM induction	Pre-infusion	Pre-sacrifice
Body mass			
CW	274.5±9.2	519.5±21.6	568.3±19.4
C-TERT	256.2±8.4	544.4±16.7	602.1±21.1
DMW	220.1±7.3	270.8±21.2*	311.0±23.6*
DM-TERT	241.4±6.3	338.1±17.3*	386.6±18.1*
Fasting glycemia			
CW	82.0±4.7	95.5±1.7	106.0±6.6
C-TERT	72.3±2.0	96.0±1.9	104.6±3.0
DMW	104.2±26.9	449.5±19.2*	451.4±24.2*
DM-TERT	85.6±7.8	361.4±36 1*	374.7±37.4*

Data are reported as means±SD (n=6-0 rats/group). *P<0.05 *vs* C group (ANOVA). CW: control rats wounded; C-TERT: non-diabetic rats wounded and infused with hBMSC-TERT; DMW: diabetic rats wounded; DM-TERT: diabetic rats wounded and infused with hBMSC-TERT.

### Wound healing evolution

**Figure 3:**
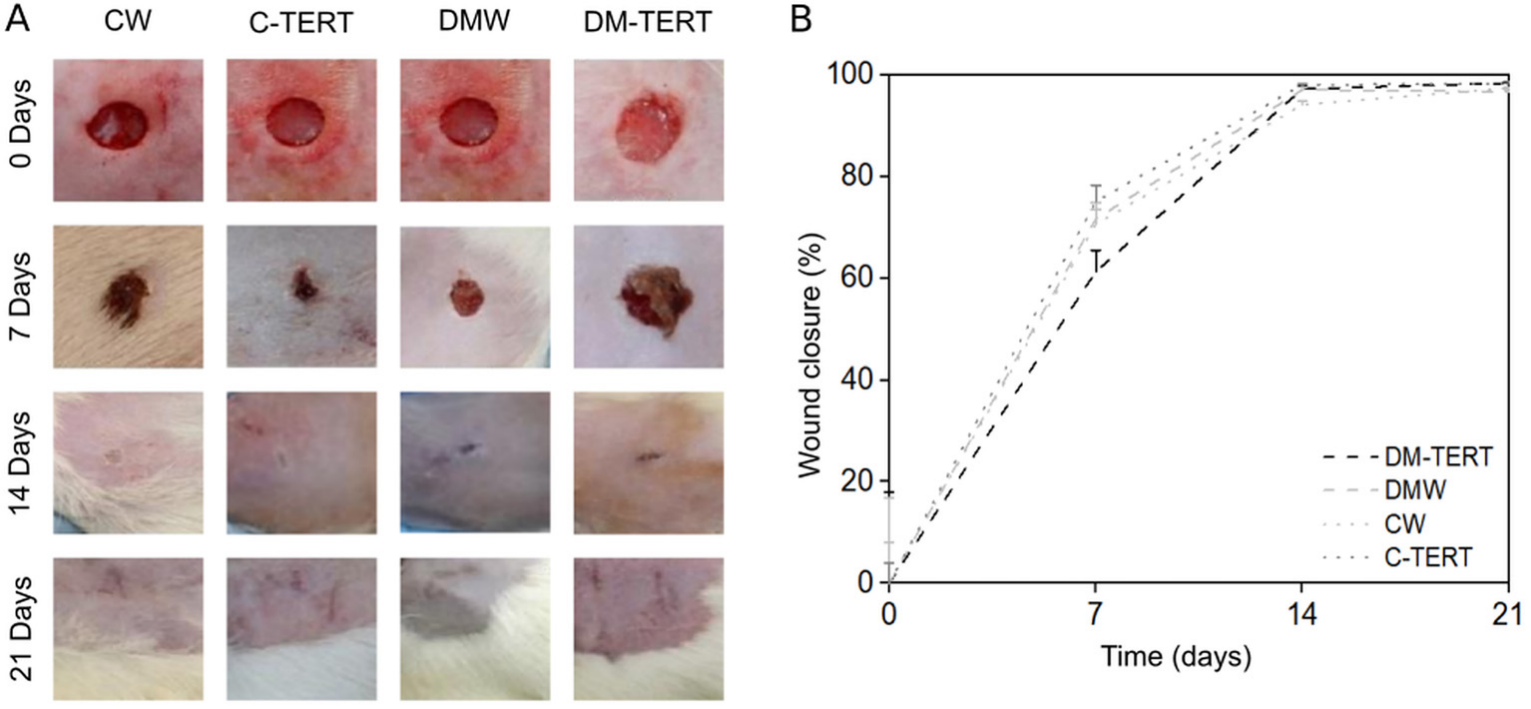
Effects of human bone marrow mesenchymal cells immortalized by the expression of telomerase (hMSC-TERT) infusion on wound closure. **A**, Representative images of wounds in rats from the groups evaluated on days 0, 7, 14, and 21. **B**, Percentage of wound reduction in the healing area on days 0, 7, 14, and 21 post-injury. The data are reported as means±SD. CW: control rats wounded; C-TERT: non-diabetic rats wounded and infused with hBMSC-TERT; DMW: diabetic rats wounded; DM-TERT: diabetic rats wounded and infused with hBMSC-TERT.

By the 14th day after wounding, all the lesions had recovered and the wound closure percentages were not different among the groups (Figure 3A and B).

### Histological analyses

**Figure 4:**
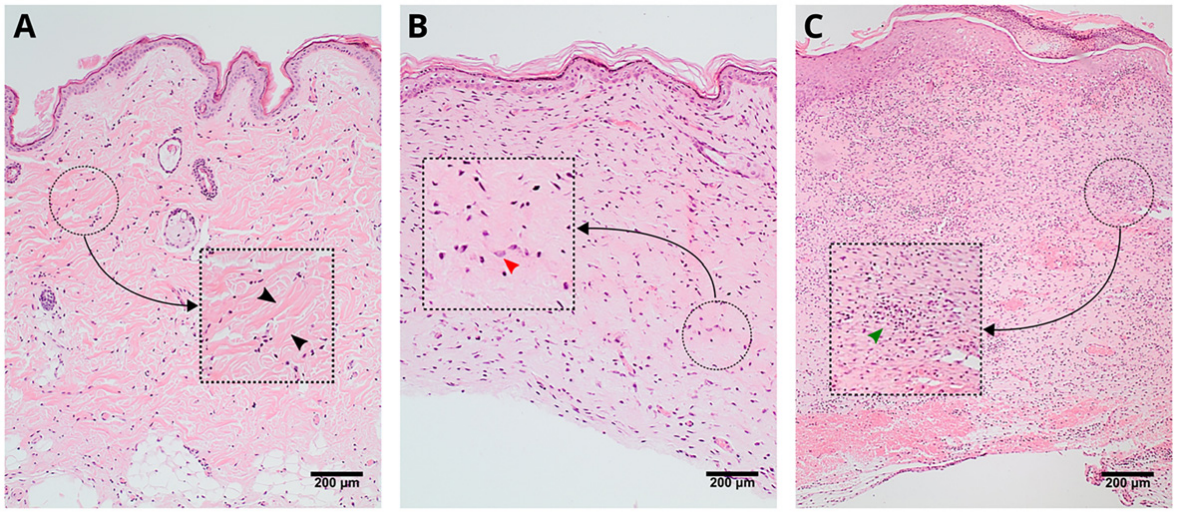
Wound histological representative images stained by hematoxylin-eosin showing a collagen fiber (black arrow) (**A**), fibroblast (red arrow) (**B**), and inflammatory infiltrate (green arrow) (**C**). Scale bar 200 μm.

**Figure 5:**
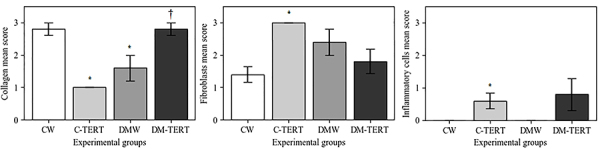
Score analysis of collagen (**A**), fibroblasts (**B**), and inflammatory cells (**C**) of skin wounds after treatment with human bone marrow mesenchymal stem cells immortalized by the expression of telomerase (hBMSC-TERT). Data are reported as means±SD for n=6-10 rats/group. *P<0.05 *vs* CW; ^†^P<0.05 *vs* DMW (ANOVA). CW: control rats wounded; C-TERT: non-diabetic rats wounded and infused with hBMSC-TERT; DMW: diabetic rats wounded; DM-TERT: diabetic rats wounded and infused with hBMSC-TERT.

Representative images of the histological sections are presented in Figure 4. We evaluated the presence of epidermal thickness, blood vessel proliferation, granulation tissue, fibroblasts, collagen, inflammatory cells, and neoplasia. Epidermal thickness and blood vessel proliferation were similar in all groups. In all samples analyzed, the epithelialization process was complete at the time the samples were collected. Mature granulation tissue was present only in the DMW group. Figure 5 shows the score values of fibroblasts, collagen, and inflammatory cells. The mean score of fibroblasts was significantly higher in the C-TERT group than in the CW group. Collagen mean score was significantly lower in the C-TERT and DMW groups than in the CW group and was higher in the DM-TERT than in the DMW group. This indicated that the CW and DM-TERT groups were in a more advanced process of tissue repair than the DMW and C-TERT groups. Regarding the inflammatory infiltrate mean score, there was no significant difference among the groups. No evidence of neoplasia was observed in any group.

### Birefringence analysis

**Figure 6:**
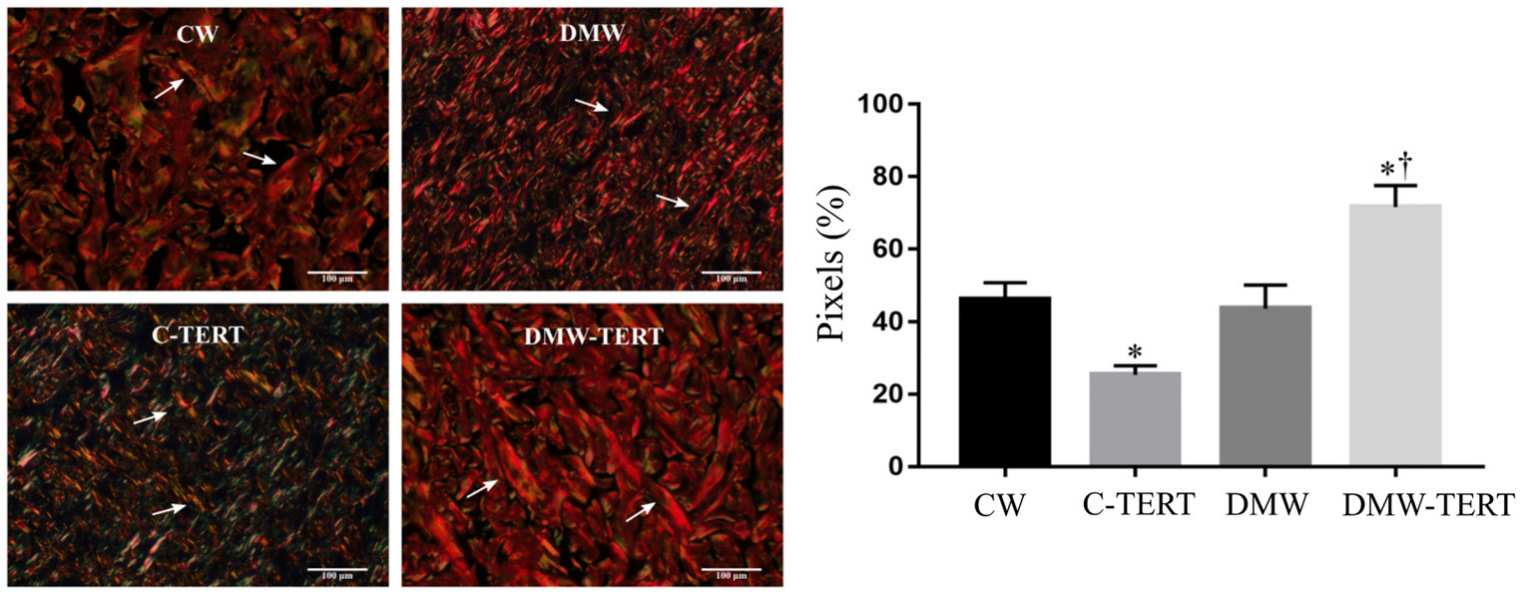
Quantitative birefringence analysis for the groups after treatment with human bone marrow mesenchymal cells immortalized by the expression of telomerase (hBMSC-TERT). White arrows indicate the birefringent fibers. Data are reported as means±SD. *P<0.05 *vs* CW; ^†^P<0.05 *vs* DMW (ANOVA). Scale bar 100 μm. CW: control rats wounded; C-TERT: non-diabetic rats wounded and infused with hBMSC-TERT; DMW: diabetic rats wounded; DM-TERT: diabetic rats wounded and infused with hBMSC-TERT.

The percentage of the collagen fibers analyzed by the picrosirius-red method was significantly lower in the C-TERT and DMW groups than in the CW group and was higher in the DM-TERT than in the DMW group (Figure 6). This pattern was similar to that obtained by the histological analysis.

## Discussion

The results of the present study demonstrated that multiple hBMSC-TERT infusions improved the wound healing process. This was the first experimental approach to investigating the use of such cells in skin tissue wounds in animals affected by DM.

In the non-diabetic condition, the healing process of skin wounds, despite being considered complex from a biological point of view, evolves in an orderly manner with the stages of inflammation, proliferation, formation of granulation tissue, and remodeling, and different molecular cascades from injured tissues are needed, which together provide the necessary incentives and release of specific cytokines and growth factors. In the case of metabolic disorder by DM, some of these biological functions are impaired and thus the healing process is impaired (28).

The mechanism by which MSCs could accelerate cutaneous wound healing may include increasing fibroblast growth and migration, skin angiogenesis, and differentiation into fibroblasts and endothelial cells (29). The trophic factors secreted by the MSCs, which exhibit immunomodulatory, anti-inflammatory, anti-apoptotic, and angiogenic effects can play an important role contributing to this tissue regeneration process, which would result in a better evolution of the healing process as a whole (30-32). Interestingly, the results of this study further indicate that the effects found are the result of an exclusively local action of this cell type by paracrine communication, since no systemic changes were observed (33,34). Paracrine activity is widely described in the literature as one of the main therapeutic effects of MSCs (34-37).

In diabetes, it has also been shown that MSCs accelerate wound healing (17). However, the application of hBMSC-TERT *in vivo* is innovative. The hBMSC-TERT cells are human mesenchymal cells immortalized by the expression of the telomerase gene (20). The ectopic expression of the telomerase hTERT gene has been shown to effectively maintain the size of telomeres that are generally shortened in cells with senescent replicative phenotype. Therefore, the expression of the gene provides an increase of the lifespan of these cells. One of the main advantages of its use is the possibility of large-scale expansion, essential to provide an adequate amount of stem cells for preclinical and clinical use. Unlike primary MSCs, that undergo senescence after a few division cycles, hBMSC-TERT have stability over long periods of expansion. Among the advantages of immortalization with TERT are the reduction of excitotoxicity and the promotion of angiogenesis. This property may be an advantage for diabetic skin healing since it has been demonstrated that glucose metabolites and by-products accelerate ageing and can reduce the functioning ability of stem-cell-based therapeutic interventions (37).

In addition, studies have shown that immortalized cells maintain their differentiation potential and do not induce the development of tumors or chromosome aberrations. However, it is important to consider that the tumorigenicity potential of hBMSC-TERT is controversial (38). Excluding the possibility of tumor formation is an essential requirement for clinical use. In the present work, the most evident contribution of the hBMSC-TERT to the healing process might have been the acceleration of the development and progression of granulation tissue. Considering granulation a physiological step of the proliferative stage along wound healing progression (37), the absence of granulation in the CW group indicated that by the end of the experimental period, a normal granulation stage could have been already past. In the DMW group, granulation tissue was still present, with different stages of maturation. Indeed, abnormally lasting granulation tissue is one characteristic of the diabetic wound (39).

On the other hand, in the DM-TERT group, granulation tissue was scarce demonstrating the acceleration of the healing process. Curiously, in the C-TERT group, granulation tissue was present, indicating that the effect of the hBMSC-TERT cells was counterproductive when interacting with normal non-diabetic wounds.

The scoring analysis is in accordance with this result. Using the scoring criteria (26), we found that the DM-TERT and CW groups had very similar patterns of fibroblastic response and collagen proliferation. This occurred in spite of differences in the inflammatory infiltrate score. Normal healing progresses from the proliferative stage, in which granulation takes place, to the remodeling stage. This last stage aims to achieve the maximum tensile strength through reorganization, degradation, and resynthesis of the extracellular matrix. It attempts to recover the normal tissue structure, and the granulation tissue is gradually remodeled, forming a scar tissue that becomes less cellular and vascular and that exhibits a progressive increase in its concentration of collagen fibers (39). The score analysis in the present work emphasized that infusions of hBMSC-TERT improved the healing process in diabetic rats (DM-TERT) to an extent closer to the normal healing process (CW) compared to untreated diabetic animals (DMW).

In addition to the action of hBMSC-TERT in the formation of granulation tissue, it is possible to emphasize its effects in the other phases of wound closure. Thus, after the inflammation phase, the proliferation phase begins followed by the remodeling phase where the structure of the new extracellular matrix is formed and the blood supply to the damaged area is reduced (39), and it is precisely in this phase that the DM-TERT group had a collagen quality comparable to the control group, which would indicate that despite the limitations of the diabetic tissue, such as the incomplete or uncoordinated healing process, therapy with hBMSC-TERT possibly allowed an expressive improvement in the conditions of the micro-environment of the wound, allowing for an adequate remodeling and healing process. Regarding collagen, several studies with stem cells show that they can boost wound healing by regulating the production and deposit of collagen in the extracellular matrix (18,33). Previous studies of our group demonstrated that stem cells derived from bone marrow improve the quality and organization of muscle collagen in diabetic rats (36). Thus, when observing the birefringence images (Figure 6), the deposition and organization of the collagen in the DM-TERT group was significantly better than the DM group and comparable to the control group. These findings allow us to conclude that, in agreement with the literature, hBMSC-TERT cells, similarly to other types of stem cells, are also capable of assisting in the synthesis and remodeling process of collagens present in the extracellular matrix of diabetic animals despite all the limitations of the micro-environmental inflammation characteristic of the disease. In addition, these cells have the advantage of not undergoing senescence after a certain number of replications, maintaining their phenotypic characteristics and potential for differentiation, and may become a therapeutic option for wounds in individuals with diabetes.
